# Using reflectance spectra and Pl@ntNet to identify herbarium specimens: a case study with *Lithocarpus*

**DOI:** 10.1111/nph.70258

**Published:** 2025-06-04

**Authors:** Barbara M. Neto‐Bradley, Pierre Bonnet, Hervé Goëau, Alexis Joly, Jeannine Cavender‐Bares, David A. Coomes

**Affiliations:** ^1^ Department of Plant Sciences and Conservation Research Institute University of Cambridge Cambridge CB23EA UK; ^2^ AMAP, University of Montpellier, IRD, CNRS, CIRAD, INRAE Montpellier 34980 France; ^3^ Inria, LIRMM, University of Montpellier, CNRS Montpellier 34095 France; ^4^ Department of Organismic and Evolutionary Biology Harvard University Cambridge MA 02138 USA; ^5^ Department of Ecology, Evolution and Behavior University of Minnesota Minneapolis MN 55108 USA

**Keywords:** herbarium specimens, indeterminate specimens, leaf reflectance spectra, *Lithocarpus*, Pl@ntNet, species identification

## Abstract

- The digitisation of plant collections is bringing large quantities of information into accessible electronic databases. However, in recent decades, traditional taxonomic work in collections has declined, meaning that more specimens are only determined to family or genus, particularly when lacking key identification structures. If unaddressed, large‐scale digitisation risks widening the gap between well‐studied species and those lacking data.
- Hyperspectral reflectance and computer vision are two emerging approaches for identifying species, but these have yet to be cross‐compared for herbarium‐based taxonomy. Using *Lithocarpus* species as a case study, we compared classification accuracy obtained from leaf reflectance spectra with computer vision (implemented via Pl@ntNet), a RGB (red, green, blue) image‐based approach known to work well on specimens presenting reproductive structures. In the spectral approach, we assessed how much data are needed to optimise classification accuracy, how many species could be discriminated between, and whether close relatives were more frequently confounded.
- We found that *Lithocarpus* herbarium specimens were accurately identified to species from relatively small spectral datasets. Despite not incorporating reproductive structures, this was only 14% less accurate than Pl@ntNet.
- We suggest these rapid, nondestructive leaf reflectance measurements, paired with computer vision, could fill identification gaps in collections, particularly for specimens lacking reproductive features.

## Introduction

**Fig. 1:**
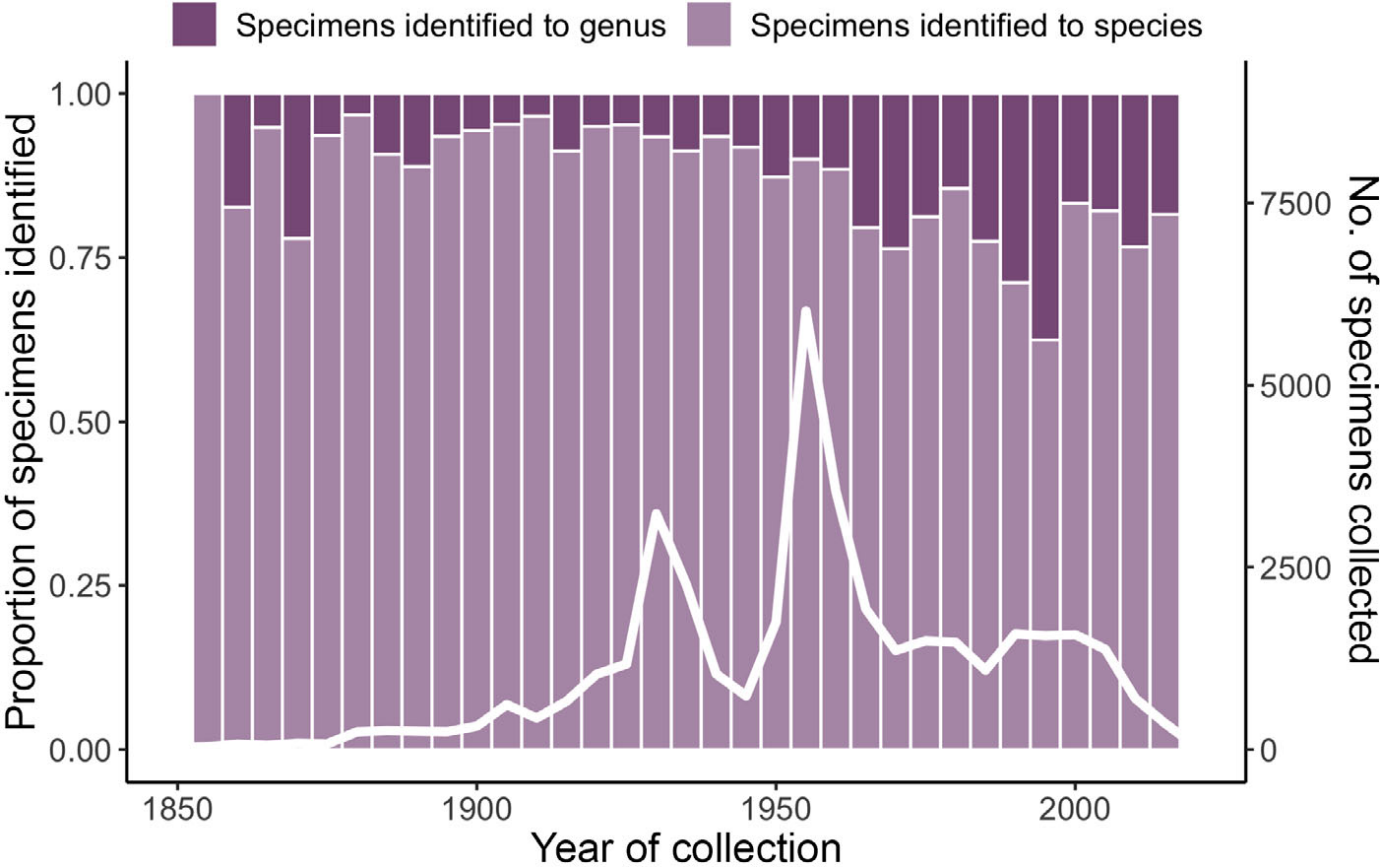
Depiction of identified herbarium specimens for species in the genus *Lithocarpus*, which were collected between 1850 and 2020. Records (downloaded from GBIF on 28 October 2024) are binned into 5‐yr intervals. The records collected in each time interval are shaded in purple, in proportion to how many have only been identified to genus vs species, shown on the left *y*‐axis. Portions of each bar shaded in dark purple represent specimens which have only been identified to genus (i.e. ‘*Lithocarpus* indet.’) and portions shaded light purple represent those identified to species. The overlaid line graph in white illustrates the number of *Lithocarpus* specimens collected in each time interval, shown on the right *y*‐axis.

Carefully curated natural history collections provide a wealth of biodiversity data, which can help us understand how species are responding to change. Long‐term, repeat observations facilitate the identification of changes in species' morphology, phenology and ranges across space and time (Knapp *et al*., 2012). Yet for many regions and species, particularly in the global south, we lack spatially and temporally stratified datasets (Yesson *et al*., 2007; Gonzalez *et al*., 2016; Meyer *et al*., 2016). In such cases, herbarium specimens from natural history collections provide an opportunity to make up for this missing biodiversity data gap faster than new fieldwork (Vargas *et al*., 2024), although we note that species from the global south are also less likely to have repeat exemplars through time. Herbarium specimens provide a snapshot of biodiversity at a particular time and place, and are increasingly recognised for their value to research. Large digitisation projects of natural history collections can enhance accessibility for researchers to leverage these specimens in addressing various ecological and evolutionary knowledge gaps. However, as most research and conservation work are organised around species‐level data, the scientific community risks overlooking a substantial portion of biodiversity in those specimens, which remain unidentified (i.e. ‘indeterminate’) in collections, despite digitisation (Lavoie, 2013; Daru *et al*., 2018; Marsico *et al*., 2020). Indeterminate specimens, those which have only been identified to the family or genus level, are common in collections (as illustrated in Fig. 1). They are often kept in collections because over the course of a specimen's lifetime, they might eventually become identified if experts exchange material or annotate specimens outside their home institution.

The limitations of herbarium data must be carefully considered to maximise the utility of large‐scale digitisation projects (Bebber *et al*., 2010; Meineke *et al*., 2018; Heberling & Mason, 2022). One fundamentally challenging aspect of using herbaria to fill knowledge gaps is that restricted curatorial resources for historically understudied species have likely already resulted in missing metadata. Limited curation, perpetuated by a decline in taxonomists and taxonomic work, can lead to the separation of plant material from records of when and where something was collected and from taxonomic analyses of the specimen, exacerbating identification gaps (Wheeler, 2014; Bik, 2017). This gap is compounded by the numerous specimens that lack reproductive structures. For many species, variation in reproductive structures provides the key morphological distinctions needed for species identification. In species that do not flower or fruit frequently, these features can easily elude collectors. For example, in remote and understudied regions, vouchers have often been collected during time‐limited forays or expeditions, where it was difficult to ensure that all species were fertile during the collection period, resulting in a significant fraction of specimens which only contain vegetative structures (Park *et al*., 2023). Large‐scale digitisation projects can risk entrenching the existing geographic and taxonomic biases in research by providing additional digital datasets for species that are already identified, well curated and better studied (Eichhorn *et al*., 2020; Feng *et al*., 2022). Nonetheless, if designed to do so, these digitisation projects also have the potential to address and correct these biases (Drew *et al*., 2017).

**Fig. 2:**
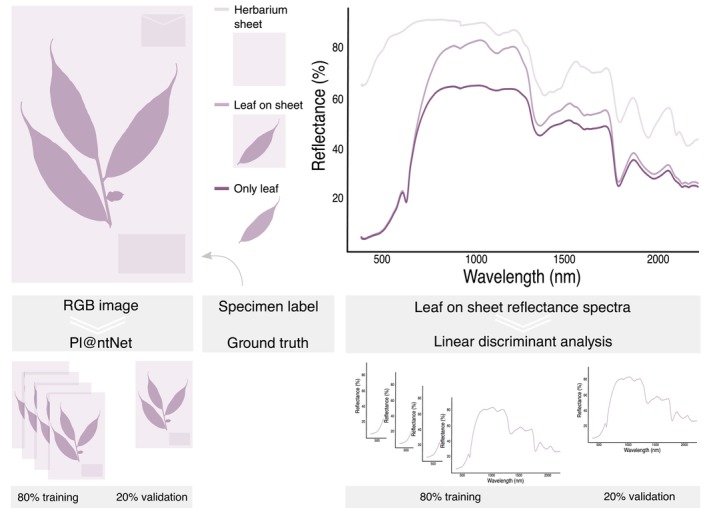
Depiction of our herbarium sampling approach and how this influences reflectance measurements. We highlight the difference between reflectance measurements from a leaf placed on a herbarium sheet (line in medium purple) vs the same leaf measured with no background (line in dark purple) in contrast to the reflectance spectra of the page itself (line in light purple). For all our analyses, we use spectra of leaf tissue measured on herbarium sheets. In our sampling design, we derived training and validation datasets for both an RGB image‐based classifier, implemented through Pl@ntNet and a spectral‐trained linear discriminant analysis (LDA) to identify species of *Lithocarpus*, using the same suite of herbarium specimens. The spectral data encode information about the chemical and physical properties of a specimen's leaves, whereas the RGB images also contain information about leaf shape and reproductive structures, when these are present.

Leaf reflectance spectra offer a complementary, nondestructive measurement for inclusion in herbarium digitisation projects, which could mitigate missing metadata. Leaf reflectance spectra are a measure of how light interacts with an object, and as such they encode the object's chemical and physical properties (Fig. 2). These nondestructive *in situ* or remotely sensed biodiversity measurements have been used to identify plant species and predict their traits from leaf spectra (Cavender‐Bares *et al*., 2016) or aerial imagery (Martin *et al*., 1998; Williams *et al*., 2021; Sapes *et al*., 2024); ongoing work highlights their potential for use with herbarium specimens (Bazan, 2024; Santacruz Endara, 2024). Kothari *et al*. (2023) showed that species identity and leaf traits are accurately estimated from spectra across fresh, dry and ground leaf tissue even when the dry material has been stored and gained discoloration. The consistent interspecific variation in leaf reflectance spectra has even been leveraged to distinguish species from their hybrid offspring with high accuracy (Stasinski *et al*., 2021). The effectiveness of spectroscopic analyses of fresh and recently dried leaves is well established (Burnett *et al*., 2021), but herbarium specimens differ from freshly dried leaves in several ways, and the efficacy of extending these approaches to include them has been underexplored (Durgante *et al*., 2013). Herbarium specimens are usually glued, taped or sewn onto sheets of paper from which they cannot be removed; they vary substantially in age (e.g. some are > 100 years old), they are collected out of convenience (e.g. from low branches and from the side of the road), and are sometimes stored for many days after collection before they are pressed (i.e. with unknown drying speeds and conditions). Herbarium specimens are limited in number and will provide relatively small training datasets for machine learning models. All these differences could make it hard to achieve reliable species identifications using spectral approaches. Consequently, evaluating the accuracy and efficacy of novel digitisation approaches for herbaria is critical before designing further work. Despite these challenges, the nondestructive nature of spectral measurements is a key advantage to be leveraged in light of the ongoing need to minimise destructive sampling of herbarium specimens and preserve them for future reference (Davis *et al*., 2024).

Machine learning approaches for identification, which leverage RGB images, are another nondestructive method that can be used to fill collection metadata gaps. The web‐ and mobile‐based platform Pl@ntNet uses advanced machine learning techniques to identify RGB images of plants with high accuracy (Joly *et al*., 2016). It was originally designed for *in situ* observations to support plant management and conservation efforts (Bonnet *et al*., 2020), but the platform has since been widely adopted by the scientific community and citizen scientists for identifying plant species through pictures of their organs (particularly, flowers, fruits and leaves), including from herbarium specimens. Its core technology is based on computer vision models, specifically leveraging deep neural networks to process vast amounts of cross‐validated plant data. At the time of writing, this includes 13 852 100 million plant images across 51 911 species. While combining and contrasting RGB classification to spectral classification is common in remote sensing, to our knowledge, this has not yet been performed in the context of identifying specimens in herbarium collections.

We focused on a subset of species in the genus Lithocarpus, as a case study for exploring the added value of measuring spectra on herbarium specimens, the sampling approach needed, and how this performs compared with RGB image‐based classification methods. We used this lineage as a case study and posit that it meets the criteria for relevant application of these methods. First, species in the genus are difficult to identify in the absence of key reproductive structures, such as fruits, but specimens are frequently collected in vegetative form (Camus, 1952; Soepadmo & van Steenis, 1972). Second, there are over 320 described species of *Lithocarpus* that span from India, East and Southward through New Guinea, and some hotspots of diversity (e.g. Mount Kinabalu) host up to 40 different species of *Lithocarpus* on the same mountain (Cannon, 2001). This high degree of sympatric species diversity underpins the need for species classification methods that do not rely on seasonal reproductive structures. To work at a scale that reflects this need, we limited our geographic focus to Malaysia, Brunei and Indonesia. We photographed and measured leaf reflectance spectra for 1467 identified specimens of *Lithocarpus* from this region, which were housed in the Herbarium at Kew (K). We then used this dataset to evaluate a sampling approach for spectra by addressing three key questions: How many measurements are needed to train accurate predictive models? How many species can be reliably distinguished in a single analysis? And does similarity in leaf traits influence identification accuracy? We then carried this forward to compare our spectral approach with an existing RGB image‐based classification method implemented through Pl@ntNet.

## Materials and Methods

### Taxonomic and geographic scope

We focused on specimens of stone oaks in the genus *Lithocarpus* (Blume) from the collections of the Royal Botanical Gardens, Kew (K) Herbarium (Richmond, UK). Specimens were selected from Kew's 6C region, which covers Brunei, Indonesia and Malaysia, with the majority of specimens originating from the island of Borneo. We prioritised looking at species that were best represented in this region. In total, we measured 1467 specimens across 31 species (specimen accessions are provided in Supporting Information Dataset S2, see later). The oldest record we observed was collected in 1850, and the youngest was collected in 2014.

### Data collection

#### Spectral data

In February 2022, we collected spectral reflectance measurements of herbarium specimens using a SVC HR‐1024i Field Spectrometer (Spectra Vista Corp., Poughkeepsie, NY, USA) equipped with a leaf clip attachment. During reflectance measurements, the gasket on the leaf clip was removed so that the spectrometer's lens could be pressed flat against the leaf tissue being measured. The spectrometer was provided and maintained by the UK's Natural Environment Research Council's Field Spectroscopy Facility (FSF, Edinburgh, UK). We measured reflectance of the adaxial surface of the leaves, avoiding major veins and visibly discoloured or damaged sections when possible. For each specimen in our dataset, we measured the reflectance of a preserved leaf glued to a herbarium sheet (as illustrated in Fig. 2). All measurements were taken on a single white‐surfaced desk, in a dark room with lights turned off and blinds drawn. Although not explored in these analyses, when more than one usable leaf was available per specimen, we measured up to three unique leaves, and for every specimen, we also measured the reflectance of the herbarium paper itself (Table S1). We calibrated measurements against a white Spectralon panel (Labsphere, North Sutton, NH, USA) every two to six measurements, between each specimen. For all spectral analyses, we use a single reflectance measurement from one leaf to represent each unique specimen. The reflectance spectra were filtered to remove readings with reflectance values > 1. We trimmed the measurements to omit noisy regions, and after cleaning, only include wavelengths between 400 and 2400. We matched the data around the sensors, using the R package spectrolab (Meireles & Schweiger, 2021). After cleaning, we smoothed, normalised and standardised the reflectance spectra before running the analyses outlined below.

#### Specimen image & metadata

We photographed each specimen using a camera mounted above a well‐lit flatbed. Specimens were placed on the flatbed alongside a ruler and colour checker card. We saved these as RGB images. For each specimen, we recorded the accession number, Latin name and collection locality. All specimens used had previously been identified, with many including determinations or confirmed identifications by Engrik Soepadmo, a taxonomist whose expertise included Lithocarpus (Soepadmo & van Steenis, 1972; Soepadmo *et al*., 2000).

### Analyses of spectral data

To assess the feasibility of using spectra to identify indeterminate herbarium species, we aimed to understand the effect of sampling on predictive power. Herbarium specimen data represent a form of convenience sampling, meaning that specimens are not always available in abundance for training identification models. As such, optimising the number of training samples relative to model accuracy was important for examining this method's transferability to smaller natural history collections. We first focused on three aspects of sampling: (1) how does model accuracy change with more training observations per species? given this; (2) how does the number of species included impact the model accuracy?; and (3) can this approach successfully discriminate between closely related species? Throughout, we used linear discriminant analysis (LDA) to predict species identity from the leaf reflectance spectra, with 80% of the data used for training and 20% for validation. Linear discriminant analysis is an established method used for spectral classification of plant species, from remote sensing to point measurements (Durgante *et al*., 2013; Guzmán *et al*., 2020; Ball *et al*., 2024). Partial least squares discriminant analysis is another common method for analysing spectral data; however, in preliminary tests, we found LDA to provide higher classification accuracy for these species with smaller training datasets (Fig. S1). We built these models using the R packages caret and mass (Kuhn, 2008; Venables & Ripley, 2010). We focused on reflectance measurements taken from the leaves mounted on the herbarium page (excluding loose leaves in packets) so that all records would be immediately comparable.

#### Effect of number of specimens per species on classification accuracy

To determine how many records are needed per species to reliably predict species identity, we focused on the three best‐represented species in our dataset (*Lithocarpus nieuwenhuisii* (Seemen) *A. Camus, Lithocarpus leptogyne* (Korth.) Soepadmo and *Lithocarpus gracilis* (Korth.) Soepadmo; Table S1). This allowed us to explore how prediction accuracy changes over a 10‐fold increase in available records, ranging from 10 to 100 records per species. All records used had been previously identified to species, enabling straightforward evaluation of prediction accuracy. We built classification models using an increasing number of observations, starting with 10 records per species and incrementing by 10 up to a maximum of 100 records. For each sample size (10, 20, 30 … 100), we repeated the model‐building process 100 times to account for uncertainty due to sampling. To quantify the relationship between the number of observations and model accuracy, we fit a segmented linear regression using the R package segmented (Muggeo & Vm, 2008). By identifying a breakpoint, we determined the least number of specimens for which we optimise improvements in classification accuracy.

#### Effect of number of species on classification accuracy

A second focus for developing an effective sampling strategy involved understanding how predictive power changed as more species were added to the dataset. To explore this, we used all species for which we had at least 30 specimens, based on our previous analysis. After filtering, the dataset contained 17 species. Using LDA as above, we evaluated the impact of increasing the number of species in the dataset on model accuracy. We built classification models using an increasing number of species, ranging from 2 to 17, and repeated this with all unique combinations of species at every sampling level. This ensured that our results were not biased by particular species combinations that are unusually easy or difficult to differentiate.

#### Effect of leaf trait similarity on classification accuracy

To assess our ability to discriminate between species even when they might share high overlap in described leaf traits, we examined the relationship between misclassification rate for each species pair in the dataset relative to their overlap in 53 leaf traits. We estimated the overlap in species' leaf traits by converting leaf descriptions from Tree Flora of Soepadmo and Saw (2000) into binary traits (e.g. elliptic leaf shape: yes/no) or numerical ranges (e.g. leaf length: 6–15 cm). For each trait, we categorised whether combinations of species were ‘distinct’ or ‘similar’. For binary traits, species pairs with a combination of 0 and 1 values were considered distinct. For continuous traits, pairs of species which had nonoverlapping ranges for the trait of interest were considered distinct. For each species pair, we then counted how many of the 53 traits were similar vs distinct, to generate an overall leaf trait similarity score. Using this approach, we populated a matrix of similarity scores for the 17 species. To assess misclassification rate, we repeated the model‐building exercise described in the Effect of number of species on classification accuracy section 100 times to incorporate variation from specimens sampled for training vs validation data. We calculated the misclassification rate as the proportion of specimens of Species A that were incorrectly identified as Species B. We then fit a linear regression, to assess whether overall leaf trait similarity was a good predictor of these misclassification rates. Finally, to identify whether specific trait differences between species were more likely to be associated with spectral classification error, we ran a redundancy analysis (RDA) with pairwise misclassification rates explained by the full matrix of pairwise trait differences between species. We also ran a similar analysis, based on phylogenetic proximity, presented in Notes S1.

#### RGB image‐based classification methods

Lastly, to contextualise the utility of approaches using reflectance spectra for classification tasks, we compared the accuracy of our spectral identification approach with that of a RGB image‐based classifier, implemented through Pl@ntNet. The model is primarily built upon the vision transformer (ViT)‐based DINOv2 architecture (Oquab *et al*., 2023), which provides robust visual embeddings for plant identification tasks (Goëau *et al*., 2023). Both models (Pl@ntNet's image‐based classifier and the spectral‐based LDA) were trained and validated using data from the same set of herbarium specimens (30 specimens per species across 17 species, split into 80% training data, 20% validation data). For the purpose of this work, Pl@ntNet has been fine‐tuned to specialise in identifying plant species from herbarium specimen images, a unique use case that differs significantly from the model's original application on fresh or living plant images. Herbarium specimens, typically consisting of pressed and dried plant material, provide valuable morphological information but present distinct challenges for machine learning due to differences in texture, colour and the presence of handwritten or printed labels (Goëau *et al*., 2021). To address this, the classification head of the Pl@ntNet model was fine‐tuned specifically for herbarium specimens while keeping the pretrained DINOv2 embeddings.

For fine‐tuning, the classification head was retrained using 24 herbarium images across 17 species. This approach preserved the strong generalist knowledge captured by the original DINOv2 embeddings, allowing the extraction of useful visual features from plant images, while tailoring the classification layers to the unique properties of herbarium specimens. By keeping the original embeddings, the fine‐tuning process benefited from the generalist nature of the DINOv2 model, which has been trained on a wide diversity of plant images, ensuring that the model retains its capability to recognise a broad array of features even in ‘less‐than‐ideal’ imaging conditions typical of herbarium collections.

## Results

**Fig. 3:**
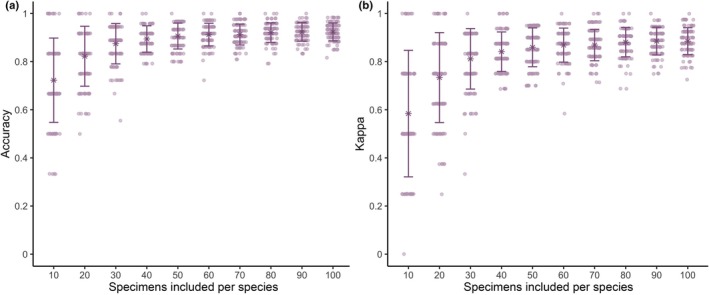
Depiction of the linear discriminant analysis (LDA) model's accuracy (a) and kappa (b), for discriminating between three species (*Lithocarpus nieuwenhuisii*, *gracilis* and *leptogyne*), across an increasing availability of specimens spectral reflectance data for model construction. At each level, 80% of the data were used to train an LDA and the remaining 20% were used for validation. An asterisk represents the mean accuracy at each level, and the error bars represent one SD from this mean.

We found that with greater numbers of specimens used for training and validating three species' discrimination, prediction accuracy for species identity increases, as does precision (Fig. 3). The mean prediction accuracy when using only 10 records per species is 0.72 (SD = 0.18) vs 0.92 (SD = 0.04) when this dataset is tenfold (Fig. 3). Over this same range, the kappa coefficient is 0.58 (SD = 0.26) and 0.89 (SD = 0.06) (Fig. 3). To identify the least number of specimens for which we would have the greatest accuracy, we fit a segmented linear regression to assess the relationship between number of specimens and accuracy and identify a breakpoint. The overall model was significant, explaining 35% of the variance in accuracy (*R* ^2^ = 0.35, *F*(1, 997) = 352.95, *P* < 0.0001). We found a breakpoint at 32 (95% CI (29, 35)) specimens. Before the breakpoint, the number of specimens had a positive relationship with accuracy (slope = 0.0076, 95% CI (0.0064, 0.0087)). After the breakpoint, this relationship remained positive but became less steep (slope = 0.00046, 95% CI (0.00016, 0.00077)). These results indicate that past 32 specimens per taxa, the added value for each specimen to our model performance decreases.

**Fig. 4:**
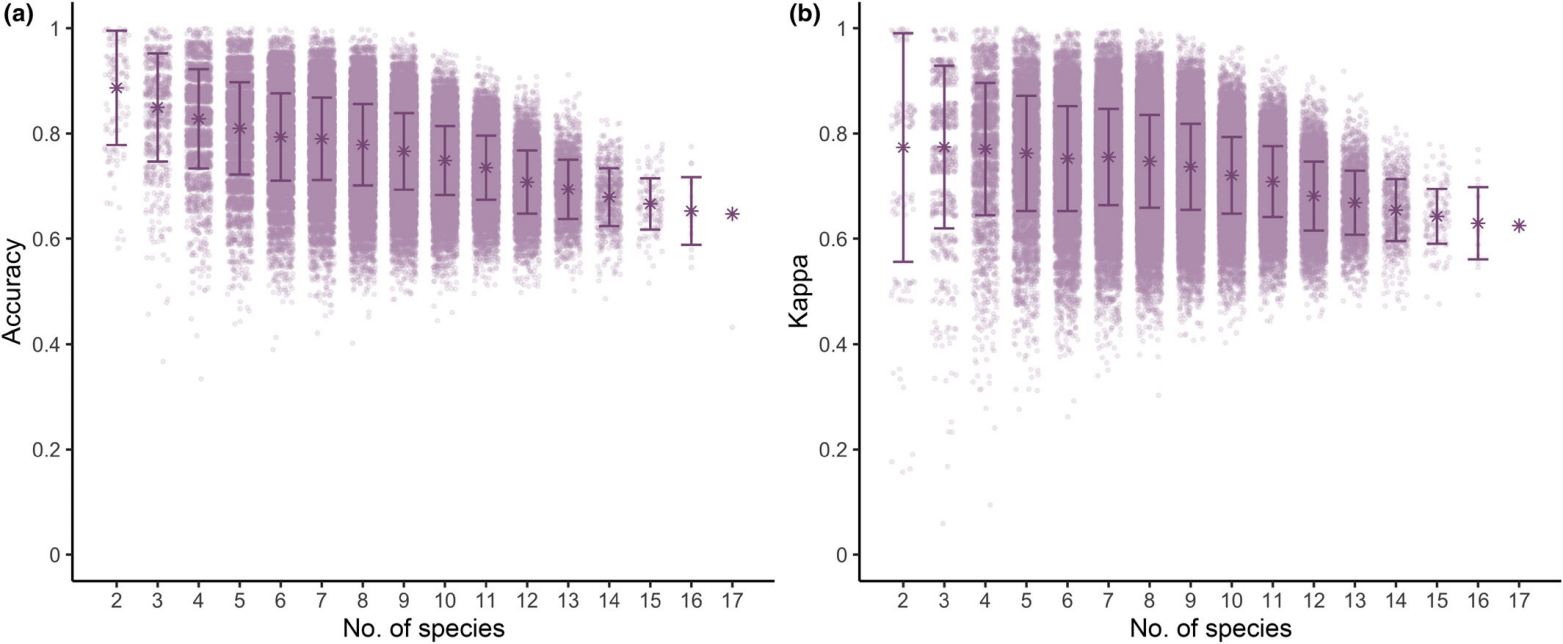
Depiction of the linear discriminant analysis (LDA)'s accuracy (a) and kappa (b) for discriminating between an increasing number of species, using 30 specimens reflectance spectra per species. To help capture the variability in model performance, for each number of taxa, all possible combinations within the full set of 17 species were considered. In each iteration, 80% of the data were used to train the model, and the remaining 20% were used to validate it. An asterisk represents the mean accuracy at each level, and the error bars represent one SD from this mean.

We then focused on all 17 species for which we had at least 30 unique specimens and evaluated the relationship between the number of species being discriminated between and model performance. As the number of species in the dataset increases, the prediction accuracy for species identity declines. The mean prediction accuracy when only discriminating two species is 0.89 (SD = 0.11) vs 0.65 (SD = 0.06) when discriminating between 16 species (Fig. 4). Over this same range, the kappa coefficient is 0.77 (SD = 0.22) and 0.63 (SD = 0.05) (Fig. 4).

**Fig. 5:**
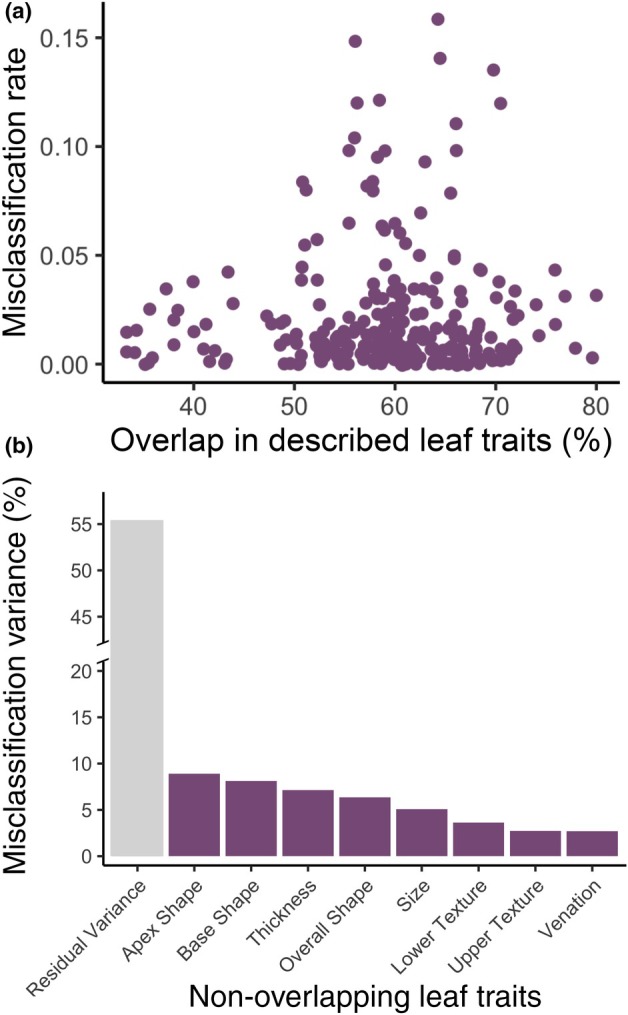
Description of the spectral misclassification between 17 species in the dataset in relation to their described leaf trait similarity. (a) Error rates between each species pair in the dataset (i.e. how frequently Species A is misclassified as Species B by the spectral model), which are plotted against the overlap in described leaf traits between each species pair. To show the full spread of variation in these error rates, every combination of taxa in the dataset is shown. (b) Variance in spectral misclassification rates, explained by differences in leaf trait categories, based on a redundancy analysis (RDA).

For these 17 species, we fit a linear regression to evaluate the relationship between misclassification rates from our spectral‐LDA and overall similarity in leaf traits. The relationship was not significant, *F*(1, 270) = 0.8551, *P* = 0.34, with the model explaining no variance in misclassification rates (Adj *R* ^2^ = 0.00, Fig. 5a). However, a RDA revealed a significant association between trait differences and misclassification, *F*(44, 227) = 4.07, *P* = 0.001, explaining 34% of the variance (Adj *R* ^2^ = 0.34; Fig. 5b). These results suggest that spectral misclassification rates are higher when species differ primarily in traits related to leaf shape and size.

**Fig. 6:**
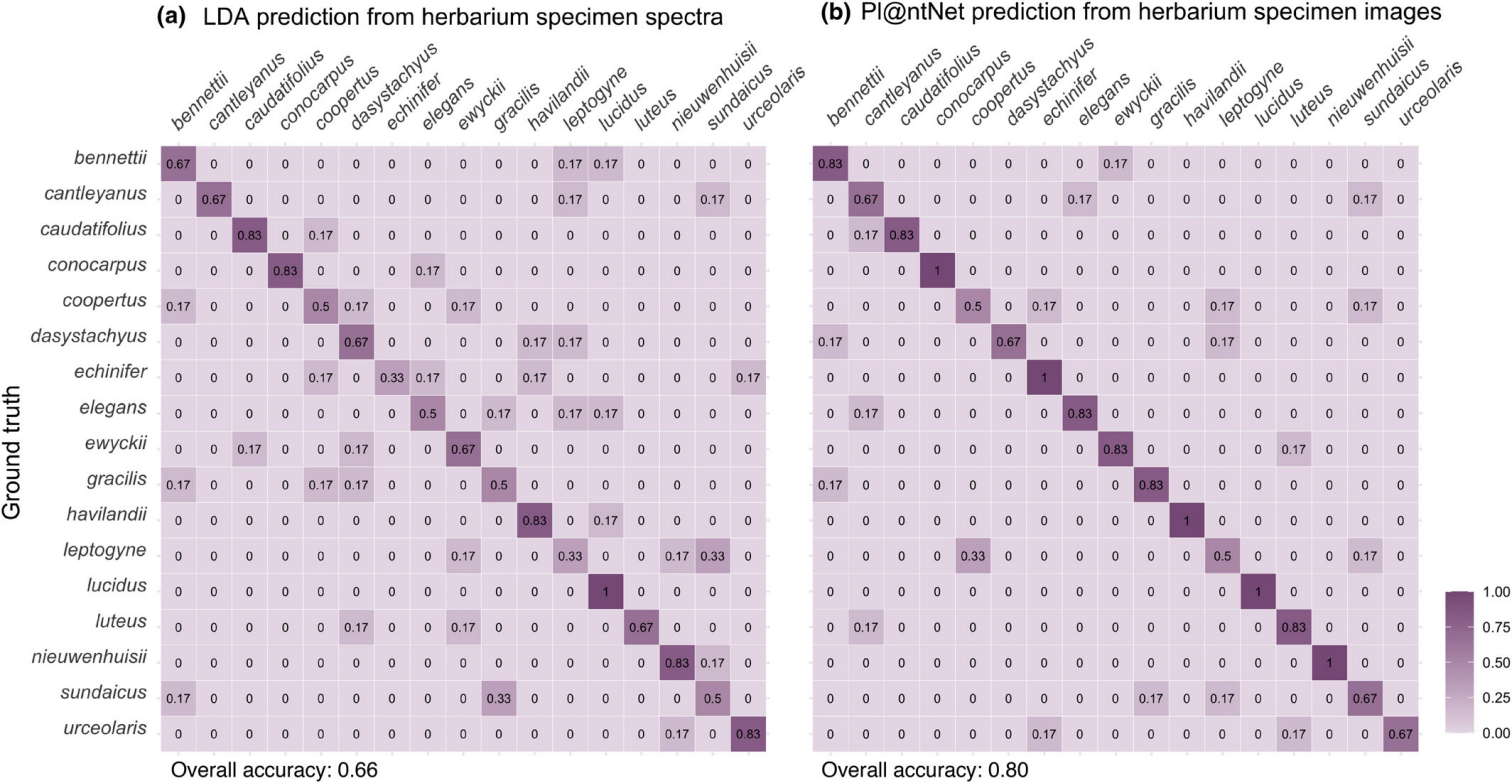
Confusion matrices for two methods of classifying 17 species of *Lithocarpus*, using the same set of herbarium specimens to train and validate both models. (a) Confusion matrix for a linear discriminant analysis (LDA) trained on spectral reflectance data from the leaves of herbarium specimens. (b) Confusion matrix for a vision transformer (ViT), implemented through Pl@ntNet, trained on whole images of each herbarium specimen. Numbers on the diagonal indicate correctly identified species; misclassifications are shown on the off‐diagonals. Darkest shades of purple highlight higher proportions of validation specimen predictions, while lighter shades illustrate smaller proportions.

**Fig. 7:**
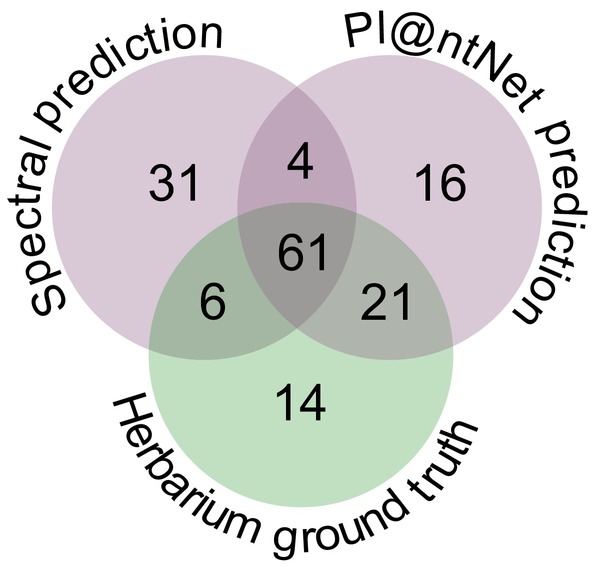
Venn diagram illustrating the overlap between species identity predictions derived from the spectral‐trained linear discriminant analysis (LDA) and an image‐trained classifier (implemented via Pl@ntNet). Both models used training (*n* = 408) and validation (*n* = 102) datasets derived from the same set of herbarium specimens. Regions of overlap with the ground truth (green circle), indicate the number of model predictions from the validation dataset, which matched the herbarium label. Regions of overlap between the spectral prediction and Pl@ntNet prediction (both purple circles) highlight validation specimens for which both models agreed in their predictions. The diagram shows that 61 individuals (out of 102) were accurately classified by both approaches, 21 species were predicted only by Pl@ntNet, six species predicted only by spectra, and 14 species were not accurately classified by either approach.

Comparing across a validation dataset of 102 specimens, the spectral‐trained LDA accurately predicted species ID for 66% of specimens, while the image‐trained model (Pl@ntNet) was 80% accurate (Fig. 6). Pl@ntNet generally had higher accuracy; however, both models made correct predictions for specimens which the other model missed (Fig. 7). As an example, Pl@ntNet was 100% accurate at identifying specimens of *Lithocarpus echinifer* where the LDA was only 33% accurate (Fig. 6). In turn, the LDA was 83% accurate at identifying specimens of *Lithocarpus urceolaris*, where Pl@ntNet was only 67% accurate (Fig. 6). Predictions for 61 specimens of 102 were in agreement across both models and matched the ground truth (Fig. 7). For 14 specimens, neither model's predictions matched the ground truth, but of these, four predictions were in agreement between both models (Fig. 7). These results suggest that both methods can bring added value to automated species classification, with key opportunities to leverage spectral classification in the absence of reproductive structures.

## Discussion

Using a realistic number of observations for the scale of a single collection, we found that rapid, nondestructive reflectance spectra measurements from herbarium specimens can be leveraged to accurately identify closely related species. Our results also demonstrate that the performance of spectral‐based classification was close to the accuracy of image‐based methods, even though the latter benefits from information provided by reproductive structures. We highlight the potential for using these approaches to identify ‘indeterminate’ specimens in collections, with spectra proving particularly valuable for specimens that lack reproductive structures.

### Relevance of *Lithocarpus* as a case study

In our study, we focused on species in the genus *Lithocarpus*, sampling those that co‐occur in Malaysia, Indonesia and Brunei, primarily on the island of Borneo. This region, known for its high biodiversity and endemism, presents a particularly challenging case for identifying species of *Lithocarpus* from herbarium specimens for multiple reasons. In particular, herbarium records show historical discrepancies in place names; many specimens were collected without fruits, despite these being key features for identification, and many specimens in collections have not been observed by an expert since before the most recent taxonomic revision (Soepadmo & Saw, 2000). These challenges make the *Lithocarpus* from Borneo an especially relevant case for evaluating our methods, as similar conditions likely apply to many understudied lineages and regions. We emphasise that this case study should be viewed as a particularly challenging scenario. We expect that prediction accuracy will improve as this field develops, and modelling approaches are designed to be particularly sensitive to the herbarium use case. We also anticipate that the accuracy achieved here will be easily surpassed when applied to more clearly defined taxonomic groups, when leveraging recently collected specimens, or when incorporating more metadata, such as spatial features.

### Sampling approaches designed for herbarium collections

While accurate species predictions depend on a sufficient number of observations regardless of the method used, collections will always have limited numbers of specimens. In this study, we justified the number of training samples needed to optimise classification accuracy by using a subset of our data to define the relationship between these, including a breakpoint analysis. To create a realistic case study for smaller herbaria, we aimed to use the fewest observations while optimising for accuracy. In our analysis, this involved 30 specimens per species, although we highlight that this number will vary depending on the taxonomic groups involved and the ease of distinguishing their subunits. We suggest that characterising this relationship and identifying the optimal sampling strategy should be independently established for each use case of these methods. As increasing the number of observations improves accuracy, we present our results as a lower bound of what can be achieved rather than a best‐case scenario.

In training our model with equal numbers of observations for each species, we avoided embedding assumptions about species' frequency in collections. With additional species‐level information, models could be trained in proportion to real‐world or collection‐level abundances. This would enable a greater proportion of specimens to be incorporated, leading to an overall greater number of observations available to include in training datasets. Further improvements in accuracy could be achieved by taking multiple spectral scans per specimen and scanning both adaxial and abaxial sides of leaves as shown in Durgante *et al*. (2013). However, as we found, this may not always be feasible depending on the condition of the material and how it is mounted on herbarium sheets. Although we gathered scans of loose leaves and the herbarium paper on its own, we did not make use of these data in these analyses. Instead we focused on scans from leaves glued to herbarium sheets, for which we had more data. We believe this is suitable for the within‐collection questions we address here, but note this may not be appropriate for aggregation with scans collected from other collections. As such, additional opportunities for improving classification accuracy might exist by ‘removing’ the effect of the herbarium paper's spectral signature from that of the leaf, or only using loose or un‐glued leaf tissue if this is possible.

### Spectral classification distinguishes between closely related species which co‐occur

Clear resolution of closely related species through spectral classification broadens the utility of this method, especially for geographic regions with high congeneric overlap or lineages that have experienced recent speciation events. Our results demonstrate that the spectral classification method does not confuse closely related species at a higher rate than more distantly related ones (Fig. S2). Our findings contribute to a growing body of infra‐generic studies (Durgante *et al*., 2013; Cavender‐Bares *et al*., 2016; Deacon *et al*., 2017; Stasinski *et al*., 2021; Santacruz Endara, 2024), which underscore the capacity to confidently resolve closely related species using spectral reflectance. In natural history collections from highly biodiverse regions, this fine‐scale taxonomic resolution will likely prove useful in making up for lacking metadata, particularly when additional data on distinguishing features, such as geographic or temporal information are missing.

As in most large tropical lineages, there is a possibility that the taxonomy of *Lithocarpus* requires revision; for example, undescribed species may potentially be housed in herbaria under other existing species names. We expect such revisions to improve classification accuracy by reducing noise in the training data rather than worsening it (Durgante, 2024). We elaborate on the opportunities to leverage spectral data in taxonomic revisions below.

### Reflectance spectra amid a broader landscape of automated classification

We show that both reflectance spectra and RGB image‐based methods offer significant value in automated species classification (Figs 6, 7). For *Lithocarpus*, as with many other species, the key distinguishing features lie in the morphology of mature fruits (Camus, 1952; Soepadmo & van Steenis, 1972). Consequently, image‐based methods that incorporate these structures will be particularly useful for classification. Despite this, the spectral method performs surprisingly, with only a 14% reduction in accuracy compared with the image‐based approach, even though it does not make use of reproductive information. This highlights the potential to confidently identify indeterminate specimens in collections that lack fruit or flowers using spectra. Our results illustrate that many species with similar leaf traits can be accurately distinguished with spectral classification (Fig. 5a). As expected, we find that classification errors are more likely when species differ primarily in leaf shape or size, features not directly captured by spectra, as opposed to texture or venation (Fig. 5b). It may be possible to mitigate some of these shortcomings of spectral‐only approaches by integrating with image‐based methods, such as Pl@ntNet.

We highlight two promising future directions that leverage both spectral and RGB image methods. First, considering the predictions of both models separately offers a way to flag potentially misidentified and mislabelled specimens with confidence, for example, where both models agree in their predictions but disagree with the herbarium label. Second, as both methods accurately predict species that the other model may miss, joint approaches that use spectral and image data together may provide a way to boost automated classification accuracy in challenging lineages. As a preliminary test, we implemented a simple approach that combines predictions from both models, weighting each based on the likelihood of accurate identification for the predicted species (Notes S2; Fig. S3). This approach is further detailed in the Supporting Information. We note several other ways of combining these approaches that should be explored.

### Opportunities from including reflectance spectra as part of the extended specimen

The importance of retrieving as much information as possible from historical specimens is growing. Natural history collections provide an efficient way to gather data on understudied taxa, compared with new fieldwork (Vargas *et al*., 2024). This can be leveraged to both understand and forecast plants' responses to climate change and other anthropogenic pressures (Feeley & Silman, 2010; Panchen *et al*., 2012; Meineke *et al*., 2019; Neto‐Bradley *et al*., 2021). Nonetheless, an estimated 30–37% of vascular plant species are represented by fewer than five observations in herbaria and ecological collections (Enquist *et al*., 2019), with small collections contributing key elements for biodiversity coverage (Marsico *et al*., 2020). This makes it crucial that we maximise the utility of existing data, particularly in regions and lineages for which it is scarce. Through our case study of the genus *Lithocarpus*, we explore how reflectance spectra can help address knowledge gaps and identify indeterminate records in collections as part of digitisation efforts. We demonstrate the effectiveness of this method even at the scale of a single collection, while noting some areas for improvement, which include refining sampling strategies and coupling spectra with image‐based classification. We hope this tool will be used to bridge the knowledge gaps for understudied species and regions as mentioned in this paper, but we also highlight that there are many exciting opportunities open for leveraging spectral data from specimens more broadly. While estimating plant traits, such as leaf chemistry, is not a new application of field‐collected spectral reflectance data (Kokaly *et al*., 2009; Serbin *et al*., 2016; Schneider *et al*., 2017), its transferability to natural history collections has been in development (Kothari *et al*., 2023). Emerging work now affirms a suite of these traits which can accurately be estimated from historical herbarium specimens using nondestructive spectral measurements (Kühn *et al*., 2024; A. K. Lee *et al*., unpublished; White *et al*., 2025). These measurements will extend what we can learn from the biodiversity time series housed in collections. In summary, we emphasise the value of spectra as part of the extended specimen, which will offer new possibilities to address questions and knowledge gaps as the field develops.

## Competing interests

None declared.

## Author contributions

BMNB, JCB and DAC designed the study. BMNB collected the data. BMNB, PB, HG and AJ analysed the data. BMNB wrote the original draft. All authors contributed to the editing.

## Disclaimer

The New Phytologist Foundation remains neutral with regard to jurisdictional claims in maps and in any institutional affiliations.

## Supporting information


**Dataset S1** Original spectral data collected for this study.

**Dataset S2** Accession list of specimens observed for this study.

**Fig. S1** Preliminary test of spectral classification with PLS‐DA vs LDA.
**Fig. S2** Classification accuracy between close relatives.
**Fig. S3** Joint spectral and Pl@ntNet classification accuracy.
**Notes S1** Effect of close relatives on classification accuracy.
**Notes S2** Using spectra & Pl@ntNet jointly for classification.
**Table S1** Summary of reflectance measurements.
Please note: Wiley is not responsible for the content or functionality of any Supporting Information supplied by the authors. Any queries (other than missing material) should be directed to the *New Phytologist* Central Office.

## Data Availability

All original spectral data are available in the Supporting Information, in the file named ‘Dataset S1’. The accession list of specimens observed is also available in the Supporting Information, in the file named ‘Dataset S2’. The code used for cleaning and analysing this dataset is available at: https://github.com/bnetobradley/lithocarpus_spectra. A public interface to Pl@ntNet is accessible at: https://identify.plantnet.org/.
