# Supplementary material for: Using reflectance spectra and Pl@ntNet to identify herbarium specimens: a case study with Lithocarpus
Source: New Phytol. 2025 Jun 4;251(2):800–10. doi: 10.1111/nph.70258 (PMC13278665; doi:10.1111/nph.70258)
Supplement: Supplementary file 3 — Fig. S1 Preliminary test of spectral classification with PLS‐DA vs LDA. Fig. S2 Classification accuracy between close relatives. Fig. S3 Joint spectral and Pl@ntNet classification accuracy. Notes S1 Effect of close relatives on classification accuracy. Notes S2 Using spectra & Pl@ntNet jointly for classification. Table S1 Summary of reflectance measurements. Please note: Wiley is not responsible for the content or functionality of any Supporting Information supplied by the authors. Any queries (other than missing material) should be directed to the New Phytologist Central Office. [file NPH-251-800-s002.pdf]

***New Phytologist* Supporting Information**

Article title: **Using reflectance spectra and Pl@ntNet to identify herbarium specimens: a case study with *Lithocarpus***

Authors: Barbara M. Neto-Bradley, Pierre Bonnet, Hervé Goëau, Alexis Joly, Jeannine Cavender-Bares & David A. Coomes

Article acceptance date: 09 May 2025

The following Supporting Information is available for this article:

Table S1 Summary of Reflectance Measurements

Figure S1 Preliminary Test of Spectral Classification with PLS-DA versus LDA

Note S1 Effect of close relatives on classification accuracy

Figure S2 Classification accuracy between close relatives

Note S2 Using Spectra & Pl@ntNet Jointly for Classification

Figure S3 Joint Spectral and Pl@ntNet Classification Accuracy

Dataset S1 Original spectral data collected for this study

Dataset S2 Accession list of specimens observed for this study

Table S1 Reflectance measurements taken for each species summarised by the number of unique herbarium specimens per species (i.e. distinct herbarium accession numbers), the number of unique leaves (n.b. a single specimen may have multiple usable leaves), and the number of loose leaves (i.e. material from herbarium packets).

Species	Unique specimens	Unique leaves	Loose leaves
Lithocarpus javensis	15	45	12
Lithocarpus beccarianus	17	42	5
Lithocarpus hallieri	10	21	2
Lithocarpus maingayi	1	2	0
Lithocarpus rotundata	1	2	0
Lithocarpus pulcher	28	54	1
Lithocarpus echinifer	38	64	0
Lithocarpus havilandii	42	79	9
Lithocarpus pallidus	5	11	1
Lithocarpus lucidus	43	64	14
Lithocarpus bancanus	23	40	0
Lithocarpus platycarpus	5	12	0
Lithocarpus indutus	10	12	0
Lithocarpus clementianus	24	33	2
Lithocarpus cantleyanus	46	106	6

Lithocarpus lampadarius	27	39	0
Lithocarpus niewenhuisii	116	232	14
Lithocarpus bennettii	62	109	9
Lithocarpus conocarpus	93	186	29
Lithocarpus ewyckii	79	149	15
Lithocarpus caudatifolius	66	139	5
Lithocarpus dachystachyus	63	117	7
Lithocarpus coopertus	90	201	37
Lithocarpus sundaicus	62	109	12
Lithocarpus elegans	55	102	6
Lithocarpus gracilis	162	304	32
Lithocarpus leptogyne	166	329	42
Lithocarpus luteus	47	93	7
Lithocarpus urceolaris	66	129	1

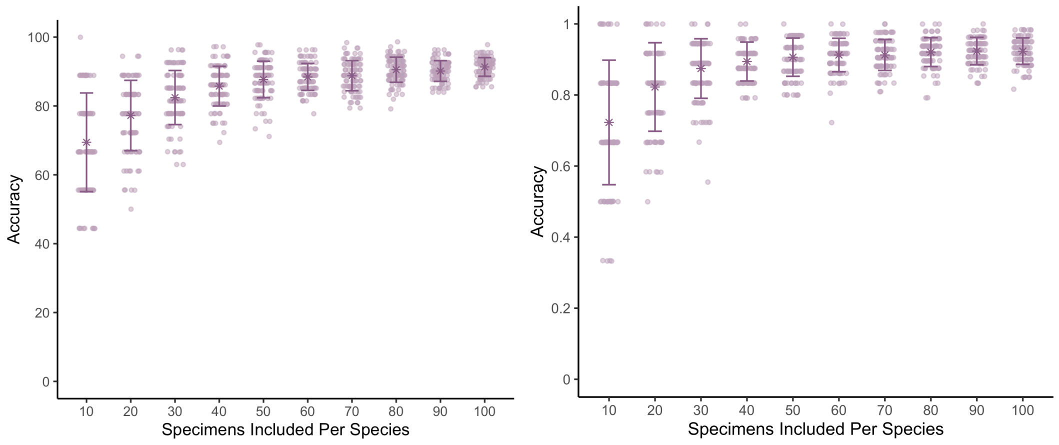

Figure S1 Illustration of 3 Lithocarpus species classification accuracy based on spectral reflectance, using partial least squares discriminant analysis (PLS-DA, left) and linear discriminant analysis (LDA, right), with an increasing number of specimens used for training these models. In this case study the LDA performs slightly better than the PLS-DA across an increasing number of observations for training. In particular, the LDA performed relatively better than the PLS-DA approach with smaller training datasets, we suggest this makes it more suitable for the herbarium sample sizes we use. An asterisk represents the mean accuracy at each level, and the error bars represent one standard deviation from this mean.

Note S1 Effect of close relatives on classification accuracy

To assess the impact of sampling closely related species on classification accuracy, we examined the relationship between misclassification rate for each species pair in the dataset and the time since those taxa last shared a common ancestor. We obtained estimates for the time since each species pair's most recent common ancestor (MRCA), from the Yang et al phylogeny for *Lithocarpus* (2018) using the r package APE (Paradis *et al.*, 2004). Since only 13 of the 17 species in our dataset were present in this phylogeny, this analysis leveraged a model built with only these 13 species, and repeated this model building exercise 100 times to incorporate variation from specimens sampled for training versus validation data. We calculated the misclassification rate as the proportion of specimens of species A that were incorrectly identified as species B. We

then fit a linear regression, with time since MRCA as the predictor for these misclassification rates.

For the 13 species included in an available phylogeny for the group, we fit a linear regression to evaluate the relationship between misclassification rates from our spectral-LDA and time since two species have shared a most recent common ancestor. We found no significant relationship, $F(1, 154) = 1.49$, $p = 0.22$, and the model explained little variance in misclassification rates ($R^2 = 0.009$). The regression coefficient for time since species' shared ancestor was not statistically significant (slope = -0.26, 95% C. I. [-0.69, 0.16], $p = .22$). These results suggest that shared evolutionary history does not predict misclassification rates in this group of species (Figure S2).

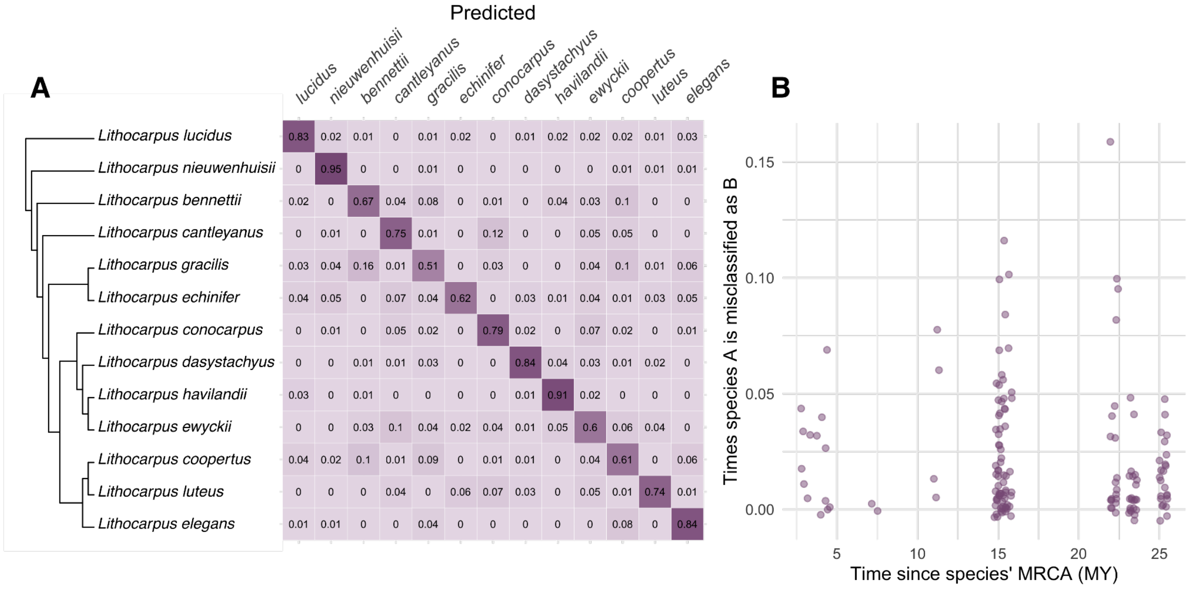

Figure S2 Depiction of the classification rates between close relatives for 13 species in the dataset. Panel A shows a confusion matrix for species identity predictions from the spectral-trained LDA, aligned with a phylogeny depicting the relationships between 13 species of Lithocarpus. Numbers on the diagonal highlight correctly identified species, misclassifications are shown on the off-diagonals. Panel B depicts the same data, as pairwise error rates between species on the y-axis (i.e. how frequently species A is misidentified as species B), plotted against the time since this species pair shared a common ancestor on the x-axis. To show the full spread of variation in these error rates, every combination of taxa in the dataset are plotted.

Note S2 Using Spectra & Pl@ntNet Jointly for Classification

We illustrate one option for jointly using both spectral and rgb-image based classification tools. The approach we use here weighs our confidence in each model's prediction when these are in disagreement. This is estimated based on the likelihood of an accurate ID given the species chosen. For example, Of 7 specimens which the spectral-LDA identified as *L bennettii*, 4 of these were accurate predictions, therefore we would weigh our confidence in future predictions of *L bennettii* from the spectral LDA by 4/7, this is then compared to our confidence in the species prediction derived from the image-based model in the same way.

Using this approach with our dataset, we find that the correct spectral predictions are disproportionately less picked up by the likelihood-weighting, and suggest this may be due to our small sample sizes resulting in coarse likelihood estimates (Figure S3). This means that the effect of one or two misclassifications can have a disproportionate effect. Larger samples may have more successful implementation of this particular joint approach, but we suggest that other joint methods need further exploration.

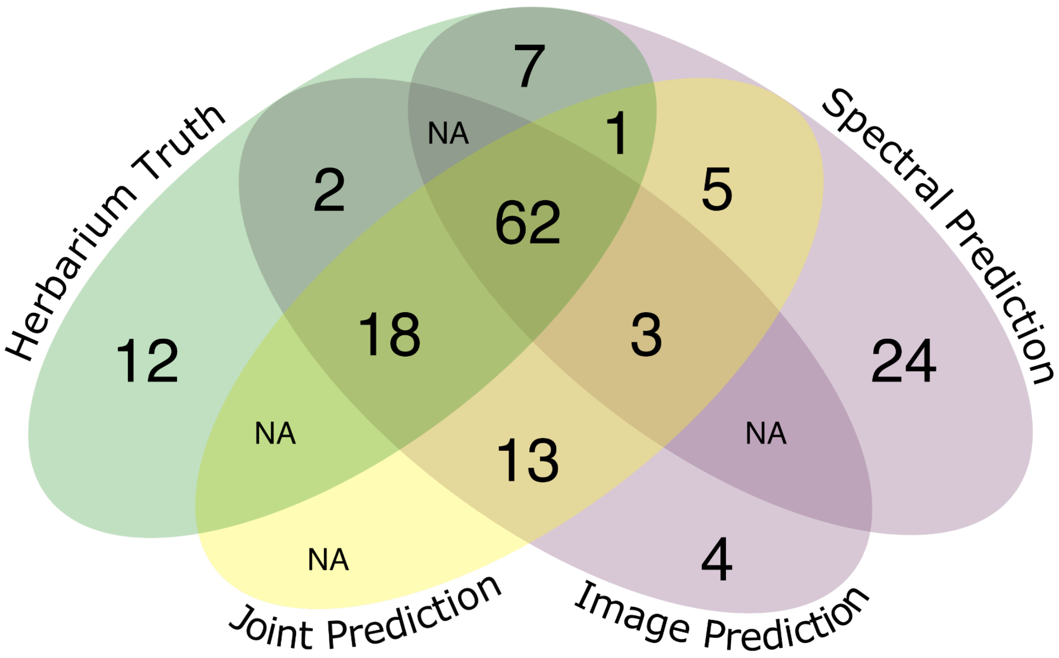

Figure S3. Venn diagram illustrating the overlap between species identity predictions derived from the spectral trained LDA, the image trained classifier, and a weighted approach using both spectral and image predictions compared with the herbarium labels (which we use as our ground truth). Both models used training (n=408) and validation (n=102) datasets derived from the same set of herbarium specimens, from which we derived likelihoods of correct identification, given species predicted. We then presented both models with a second, new validation dataset (n=102), to generate a new set of predictions, which we combined into a joint approach, by using our estimated likelihoods of correct identification to weigh both model predictions when they disagreed with each other. Regions of overlap with the ground truth (green ellipse) indicate the number of model predictions from the validation dataset which matched the ground truth. Regions of overlap between the spectral prediction and image prediction (both purple ellipses), highlight validation specimens for which both models agreed in their predictions. Regions of overlap with the yellow ellipse indicate predictions generated through the likelihood weighted approach.

References

Paradis E, Claude J, Strimmer K. **2004**. APE: Analyses of Phylogenetics and Evolution in R language. *Bioinformatics* **20**: 289–290.

Yang C-K, Chiang Y-C, Huang B-H, Ju L-P, Liao P-C. **2018**. Nuclear and chloroplast DNA phylogeography suggests an Early Miocene southward expansion of Lithocarpus (Fagaceae) on the Asian continent and islands. *Botanical studies* **59**: 27.
